# Determination of double bond positions in unsaturated fatty acids by pre-column derivatization with dimethyl and dipyridyl disulfide followed by LC-SWATH-MS analysis

**DOI:** 10.1007/s00216-024-05542-z

**Published:** 2024-10-05

**Authors:** Matthias Olfert, Cornelius Knappe, Adrian Sievers-Engler, Benedikt Masberg, Michael Lämmerhofer

**Affiliations:** Institute of Pharmaceutical Sciences, Pharmaceutical (Bio-)Analysis, University of Tübingen, Auf der Morgenstelle 8, 72076 Tübingen, Germany

**Keywords:** Lipidomics, Data-independent acquisition (DIA), Isomer, Dimethyl disulfide (DMDS), 2,2′-Dipyridyldisulfide (DPDS), Collision-induced dissociation (CID)

## Abstract

Comprehensive in-depth structural characterization of free mono-unsaturated and polyunsaturated fatty acids often requires the determination of carbon-carbon double bond positions due to their impact on physiological properties and relevance in biological samples or during impurity profiling of pharmaceuticals. In this research, we report on the evaluation of disulfides as suitable derivatization reagents for the determination of carbon-carbon double bond positions of unsaturated free fatty acids by UHPLC-ESI-QTOF-MS/MS analysis and SWATH (sequential windowed acquisition of all theoretical mass spectra) acquisition. Iodine-catalyzed derivatization of C = C double bonds with dimethyl disulfide (DMDS) enabled detection of characteristic carboxy-terminal MS2 fragments for various fatty acids in ESI negative mode. The determination of double bond positions of fatty acids with up to three double bonds, the transfer of the method to plasma samples, and its limitations have been shown. To achieve charge-switching for positive ion mode MS-detection, derivatization with 2,2′-dipyridyldisulfide (DPDS) was investigated. It enabled detection of both corresponding characteristic omega-end- and carboxy-end-fragments for fatty acids with up to two double bonds in positive ion mode. It provides a straightforward strategy for designing MRM transitions for targeted LC–MS/MS assays. Both derivatization techniques represent a simple and inexpensive way for the determination of double bond positions in fatty acids with low number of double bonds. No adaptation of MS hardware is required and the specific isotopic pattern of resulting sulfur-containing products provides additional structural confirmation. This reaction scheme opens up the avenue of structural tuning of disulfide reagents beyond DMDS and DPDS using reagents like cystine and analogs to achieve enhanced performance and sensitivity.

**Graphical Abstract:**

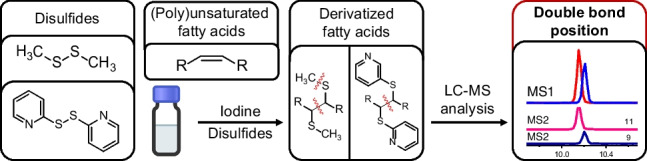

**Supplementary Information:**

The online version contains supplementary material available at 10.1007/s00216-024-05542-z.

## Introduction

Fatty acids are a diverse group of molecules and the main building blocks for lipids in the human body [1]. They are typically characterized by their carbon number and their degree and position of unsaturation. While carbon number and degree of unsaturation are readily obtained by high-resolution mass spectrometry, the determination of the carbon–carbon-double bond (DB) position represents an analytical challenge due to the isobaric nature of such constitutional isomers and limited fragmentation in MS2 of commonly employed collisional induced dissociation (CID) [2, 3]. Knowledge of DB positions, however, is of prime importance, as they have a major influence on the physiological and pathophysiological properties of fatty acids. (Poly-)Unsaturated fatty acids of physiological interest can be differentiated in ω-3-, ω-6-, and ω-9-fatty acids. From biological viewpoint, the balance of ω-3- and ω-6-fatty acids plays a critical role in the prevention of several diseases [1, 4, 5].

Structural lipidomics requires the full elucidation of molecular characteristics including DB positions. The complete structural elucidation is also necessary to get a deeper understanding regarding physiological properties of lipids but also as part of the identification of unknowns in the pharmaceutical and food industry. During quality control of starting materials and finished lipid-containing products, unspecified fatty acid isomers could be considered as impurity and proper identification is required once the respective threshold is exceeded [6, 7].

Numerous methods have been reported recently for the determination of DB positions. For instance, for GC–MS (gas chromatography-mass spectrometry) analysis, the derivatization with dimethyl disulfide (DMDS), enabling characteristic cleavage of the derivatized C–C bond by electron ionization (EI), was suggested [8–10]. Its applicability has been shown especially for mono-unsaturated fatty acids, but also polyunsaturated fatty acids (PUFAs) can be derivatized efficiently by using lower temperatures [11]. This approach involved also derivatization of free fatty acids to, e.g., methylesters or amides as an additional step in the sample preparation to achieve volatility for GC–MS analysis [12]. There is a certain risk that the required derivatization conditions alter the sample composition, especially in multi-component samples.

For liquid chromatography (LC)-MS analysis, various derivatization approaches for DB position determination have been established. For a brief overview, the reader is referred to recent reviews on this subject [13–17]. One of the most commonly used methods appears to be Paternò-Büchi reaction [18] and modified versions [19]. The original method is based on the formation of oxetane rings at the original DB position by addition of acetone during in-source online derivatization with nano-ESI-tip and activation with UV light [18]. This type of reaction can be used as a platform technology tuned with different ketones/aldehydes to achieve higher yields, fewer side reactions (e.g., for trifluoroacetophenone) [13, 20], and detection in positive ion mode by charge-switching (e.g., in-source derivatization with acetylpyridine) [13, 21]. Besides these in-source derivatizations, also “pre-source” (e.g., with inline bare fused silica capillary) [22] and pre-column derivatizations [20] have been proposed. The derivatization allows fragmentation by collision-induced dissociation (CID), generating two characteristic MS2 fragments by cleavage at the original DB position [18]. Downsides of this method includes specific hardware requirements (e.g., low-pressure mercury lamps, fused silica capillaries) and possible generation of side products and positional isomers, leading to more complex chromatograms if performed pre-column [13, 20]. The application of Paternò-Büchi reaction has been shown to be effective as part of double derivatization techniques [23] and in combination with ion mobility MS analysis [24]. Other derivatization approaches involve the introduction of stable epoxides at DB positions with low-temperature plasma [25], mCPBA (meta-chloroperoxybenzoic acid) [26, 27], oxone (potassium peroxymonosulfate) [28] or urea hydrogen peroxide [29], aza-Prilezhaev aziridination [30], derivatization with *N*-Alkylpyridinium [31], and derivatization of the carboxylic function inducing alkyl chain fragmentation with characteristic product ions allowing to pinpoint the DB position [32]. In a modified version of the mCPBA derivatization, an analytical strategy for relative quantification of FAs, carbon–carbon double bond localization, and *cis-*/*trans-*geometry differentiation was proposed by isobaric multiplex labeling reagents for carbonyl-containing compound tag conjugation and *m*-CPBA epoxidation (isobaric tagging) [33]. The typical Diels–Alder reaction has been utilized for conjugated FAs [34], while Cerrato et al. recently described the inverse-electron-demand Diels–Alder reaction for positional determination of isolated DBs in free fatty acids and other lipids [35]. Various ozonolysis-based approaches for DB localization have been proposed, in which the formed ozonides decomposed into characteristic fragments; such ozonolysis reactions have been performed off-line [36], in the ion source (OzESI-MS) [37], and in the collision cell (ozone-induced dissociation, ozID) [38]. Other gas-phase approaches include the use of electron-activated dissociation (EAD) [39, 40] and ultraviolet photo-dissociation (UVPD) instead of CID for fragmentation [15].

Our research focused on extending the analyst’s toolbox for determination of DB positions in unsaturated fatty acids. First, the applicability of the DMDS derivatization protocol for LC–MS analysis of fatty acids was evaluated for the first time. Second, the suitability of disulfide reagents as a tunable platform for DB position determination in lipids was investigated to elucidate whether (i) ionizable residues allow charge reversal, leading to detectability in positive ion mode and thus improving ionization efficiency; (ii) confirmative product ions of both ω- and carboxy-terminal ends can be designed into the concept; and (iii) such confirmative product ions from both sides of the double bond would be suitable to design *in silico* MRM transitions for targeted HPLC-QqQ-ESI–MS/MS analysis.

## Experimental

### Materials

Mass spectrometry-grade (Rotisolv, Ultra LC–MS grade) methanol, acetonitrile, and n-hexane were purchased from Carl Roth (Karlsruhe, Germany). Dimethyl disulfide (DMDS), 2,2′-dipyridyl disulfide (DPDS), palmitoleic acid (PAL, C16:1n-7), oleic acid (OA; C18:1n-9), cis-vaccenic acid (VA; C18:1n-7), linoleic acid (LA; C18:2n-6,9), γ-linolenic acid (GLA; C18:3n-6,9,12), iodine, diethyl ether, and sodium thiosulfate were obtained from Sigma-Aldrich (Merck, Taufkirchen, Germany). α-Linolenic acid (ALA; C18:3n-3,6,9), ricinenic acid (9Z,11Z-CLA; C18:2n-7,9), 10E,12Z-octadecadienoic acid (10E,12Z-CLA; C18:2n-6,8), and “Polyunsaturated Fatty Acid MaxSpec LC–MS Mixture” were from Cayman Chemical Company (Ann Arbor, USA). Commercial plasma (NIST SRM 1950) was used for the tests on the applicability to real samples and purchased from Sigma-Aldrich (Merck). Ultrapure water was produced by additional purification of demineralized water using an Elga LabWater Ultra purification system (Celle, Germany). Acetic acid (Rotipuran > 98%) was obtained from Carl Roth (Karlsruhe, Germany). Nomenclature and abbreviations of the fatty acids in accordance with LIPID MAPS [41] and Lipidomics Standard Initiative [42] can be found in Table S1.

The reversed-phase column Kinetex C8 (3.0 × 50 mm, 2.6 µm, 100 Å) was obtained from Phenomenex (Aschaffenburg, Germany).

### Instrumentation and software

The HPLC system consisted of an Agilent 1290 Infinity series UHPLC system with a thermostated column compartment and binary pump (Agilent Technologies, Santa Clara, USA) and a PAL HTC-xt autosampler (CTC Analytics AG, Zwingen, Switzerland). The HPLC system was hyphenated by a diverter valve and AB Sciex CDS (calibrant-delivery-system) to a Sciex TripleTOF 5600 + system with DuoSpray source for electrospray ionization from Sciex (Framingham, MA, USA). The system was controlled by Analyst TF 1.8.1 and the data evaluation was performed with PeakView 2.2 and Multiquant 3.0. OriginPro 2022 (version 9.9) from OriginLab Corporation (Northampton, USA) was used for data visualization. Graphical abstract was created with Chemix (https://chemix.org).

### Derivatization method

The derivatization method followed previous works with DMDS for GC–MS with some modifications [43, 44]. The following solutions were prepared: fatty acids in n-hexane (50 µg/mL), iodine in diethyl ether (60 mg/mL), DMDS as neat solution, DPDS in n-hexane (3 mg/mL), 10% sodium thiosulfate in water. The samples were prepared by mixing 200 µL fatty acid solution with 200 µL iodine solution and 400 µL DMDS or DPDS solution. The incubation was carried out at 35 °C and various time points (figures were created at the apex of the kinetic curves). To remove the excess iodine and stop the reaction, the sodium thiosulfate solution was added until discoloration of the hexane phase. After removing the hexane phase into a new vial, n-hexane was added to the sample and shaken to extract remaining fatty acids. Again, the hexane phase was pipetted into the new vial. The combined hexane fractions were evaporated in a Genevac EZ-2 evaporator (Ipswich, UK) under nitrogen and reconstituted with methanol to achieve a concentration of 1 µg/mL for LC–MS measurements. The workflow is schematically depicted in Fig. S1.

### LC–MS method

Kinetex C8, 3.0 × 50 mm, 2.6 µm was used as column. Mobile phase A consisted of water with 0.1% (v/v) acetic acid and mobile phase B of acetonitrile with 0.1% (v/v) acetic acid. The column temperature was set to 40 °C and the flow rate to 0.5 mL min^−1^. The following gradient profile was applied: 10 to 100% B in 13 min, 100% B for 3 min, 100 to 10% B in 0.1 min, 10% B for 3.9 min. The first 5 min was directed into the waste by a diverter valve.

Mass spectrometer settings were as follows: curtain gas: 30 psi, source gas 1: 50 psi, source gas 2: 40 psi, ion-spray voltage floating: − 4500 V (negative mode), 5500 V (positive mode), ion source temperature: 550 °C, declustering potential: − 80 V (negative mode), 80 V (positive mode).

Data were recorded by data-independent acquisition with SWATH technology: TOF–MS full scan with 200 ms accumulation time, scan range from *m/z* 50–1000, collision energy of − 5 V (negative mode), SWATH-MS accumulation time of 50 ms per window, and collision energy of − 45 V (negative mode). The design of the SWATH windows can be found in the Supplementary information (Table S2).

Targeted product ion experiments (MRM-HR) consisted of a TOF–MS full scan experiment with 200 ms accumulation time, scan range from *m/z* 50–1000, and collision energy of − 5 V (negative mode) and 5 V (positive mode), respectively. Collision energy was screened for all single standards from − 20 to − 60 V in negative ion mode (DMDS) or 20 to 60 V in positive ion mode (DPDS) in 5 V steps, whereas 45 V (positive ion mode) and − 45 V (negative ion mode) showed the best results. Subsequently, product ion scans were performed with an accumulation time of 100 ms and collision energy of − 45 V (negative ion mode) and 45 V (positive ion mode).

## Results and discussion

### Double bond derivatization with disulfides

The reaction of disulfides, like dimethyl disulfide (DMDS), with the double bond needs iodine catalysis. Iodine initially forms an iodonium ion by electrophilic addition to the double bond. The resulting iodide anion reacts in a nucleophilic substitution with the DMDS, forming thiomethyl anions, which react in an anti-addition with the iodonium ion. The resulting sulfonium ring is further attacked by thiomethyl anions yielding bis-methylthio derivatives at the initial DB position. This mechanism has been recently proposed by Richter et al. [45] and can be found in Fig. S2. The bis-methylthio substituents activate the C–C bond corresponding to the original DB for fragmentation by CID affording characteristic fragments for pinpointing the DB location in the unsaturated lipid. The generation of characteristic fragments in LC–MS was also shown by Deng et al. for mono-methylthio derivatives of diacylglycerides, although the exact chemistry of the formation of mono-methylthio derivatives remains unclear and could not be confirmed in our study [46].

Limitations of DMDS in LC–MS based fatty acid DB position determination include restriction to negative mode, detection of solely carboxy-terminal fragments, and lack of corresponding confirmative fragments from ω-terminal tail. To overcome this, a charge-switching concept in which the methyl groups of the disulfide were exchanged for an ionizable group, like pyridines, to enable detection in positive mode was envisaged by use of 2,2′-dipyridyl disulfide (DPDS) as derivatization reagent. It was envisioned that this concept allows extension of DB position determination to volatile analytes such as aldehydes which are difficult to analyze by LC–MS without proper derivatization [47].

Various MUFAs and PUFAs were selected as model lipids to systematically study the reactivity and reaction products as well as product ions formed. For every single standard (DMDS- or DPDS-derivatized) in each mass spectrometric cycle, the following MS experiments were performed: (i) time-of-flight (TOF)–MS scan for MS1-precursor measurement; (ii) PIS (product ion scans, MRM-HR) of the mono-derivatized product for fragmentation and recording of the respective MS2 spectra. Hereby, it was possible to simultaneously detect mono-derivatized products for DMDS and DPDS and bis-derivatized and cyclic products for DMDS on MS1 level. The summarized experimentally observed fragments for DMDS can be found in Table S3 and suggested transitions for targeted MRM-experiments in Table S4. For DPDS the respective fragments and transitions can be found in Table S5 and Table S6.

### Oleic acid (C18:1n-9) and vaccenic acid (C18:1n-7)

**Fig. 1:**
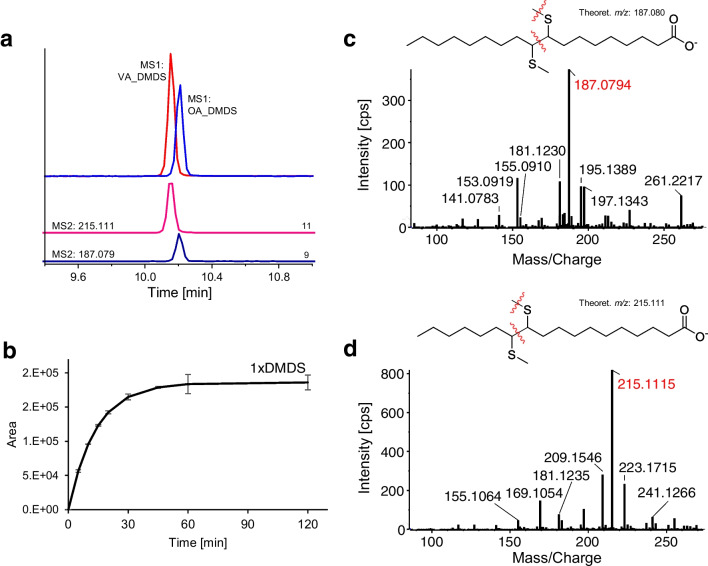
DMDS-derivatized mono-unsaturated fatty acids: **a** MS1-EIC of derivatized oleic acid (blue; *m/z* 375.240 ± 0.01) and cis-vaccenic acid (red; *m/z* 375.240 ± 0.01) from TOF–MS scans, MS2-EIC of characteristic fragments (*m/z* 215.111 ± 0.01, *m/z* 187.080 ± 0.01; intensity multiplied with 20 for better visibility) from PIS (MRM-HR) of respective mono-derivatized products; **b** derivatization kinetics of mono-derivatized oleic acid (fitted, pseudo 1st order, BoxLucas1); **c** MS2 spectrum (CE: 45 V) of derivatized oleic acid with tentative fragmentation pattern leading to fragment with *m/z* 187.080; **d** MS2 spectrum (CE: 45 V) of derivatized cis-vaccenic acid with tentative fragmentation pattern leading to fragment *m/z* 215.111

Oleic acid and vaccenic acid were selected as model MUFAs with different DB positions. Initial experiments were performed with individual standard solutions. As shown in Fig. 1a, DMDS successfully reacted with both MUFAs yielding a significant peak of mono-DMDS-derivatized oleic and vaccenic acid (yielding bis-methylthio derivatives) by negative ESI ion mode LC–MS. DMDS-derivatized vaccenic acid (VA) with double bond position at C11 (Δ11; ω7) elutes before oleic acid (OA) with its double bond at C9 (Δ9; ω9). It is striking that there is no separation of diastereomers (2 chiral centers, 4 stereoisomers) that obviously coelute which is favorable from viewpoint of sensitivity. The slight shift in retention time between VA and OA can be explained by the hindered tight interaction between the alkyl strands of C18 phase and analyte due to the methylthio substituents like in branched chain fatty acids. In the case of the oleic acid, a longer alkyl tail can still tightly interact with the C18 chain leading to stronger retention. Next, the derivatization kinetics was measured over 120 min (Fig. 1b). The peak area for the oleic acid derivative steeply increased exponentially until a plateau was reached after approximately 45 min. A pseudo-first-order rate model provided a good fit (reaction rate constant *k*: 0.0723 min^−1^, half time *t*_1/2_: 9.58 min). Differentiation between the constitutional isomers (OA and VA) is possible on MS2 level based on the characteristic carboxy-terminal fragments obtained by C–C bond cleavage and additional demethylation of the thioether (OA: *m/z* 187.080; VA: *m/z* 215.111, Fig. 1c and d). The fragment with *m/z* 215.111 was negligible in oleic acid, especially when compared to the fragment *m/z* 187.080. The correct assignments can be confirmed by other additional fragments with smaller intensities (*m/z* 153.092, 213.095 for oleic acid or *m/z* 243.142, 241.127 for vaccenic acid) which, however, are all related to the carboxy-terminal head that carries the negative charge (see Figs. S3 and S4). DMDS-derivatized palmitoleic acid (C16:1n-7), which shows a double bond in position 9, exhibits the same fragmentation behavior as oleic acid with the product ion of *m/z* 187.080 being the most intensive characteristic fragment (Figs. S5 and S6). The successful implementation of DMDS derivatization to the above-discussed MUFAs confirms the general applicability of this double bond reagent for LC–MS and CID fragmentation.

**Fig. 2:**
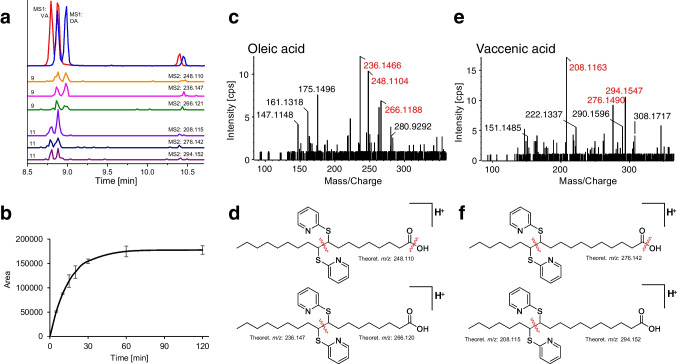
DPDS-derivatized mono-unsaturated fatty acids, detected as [M + H]^+^: **a** MS1-EIC of derivatized oleic acid (blue; *m/z* 503.276 ± 0.01) and cis-vaccenic acid (red; *m/z* 503.276 ± 0.01) from TOF–MS scans, MS2-EICs of characteristic fragments (40-fold multiplied for visibility; derived from OA: *m/z* 248.110, 236.147, 266.121; derived from VA: *m/z* 208.115, 276.142, 294.152) from PIS (MRM-HR) of respective mono-derivatized products; **b** derivatization kinetics of mono-derivatized oleic acid (fitted, pseudo 1st order, BoxLucas1); **c** MS2 spectrum (CE: 45 V) of derivatized oleic acid; **d** tentative fragmentation patterns of oleic acid; **e** MS2 spectrum (CE: 45 V) of derivatized cis-vaccenic acid; **f** tentative fragmentation patterns of cis-vaccenic acid

All experiments with 2,2′-dipyridyl disulfide (DPDS) were performed in positive ion mode. In Fig. 2a, the MS1-EICs of mono-derivatized oleic acid (OA) and vaccenic acid (VA) show for both fatty acids a major double peak between 8.5 and 9 min and one minor peak eluting significantly later than the two major peaks (at around 10.5 min). However, only the first two peaks have an MS2 spectrum which can be assigned to the derivatized fatty acids. The double peak can be explained by the formation of diastereomers which are chromatographically separated. This is unfavorable since the signal intensity is divided into two peaks and increases complexity of the chromatogram. The derivatization kinetics (Fig. 2b; reaction rate constant *k*: 0.0681 min^−1^, half time *t*_1/2_: 10.18 min), under the same MS conditions (as DMDS), is comparable to the one from oleic acid and DMDS (Fig. 1b), but the MS2 spectra show reduced intensities of the fragments compared to the derivatization with DMDS (mobile phase was optimized for DMDS derivatives in negative ESI mode but not specifically for DPDS derivatives in positive ESI mode). Nevertheless, characteristic product ions obtained by cleavage of the C–C bond, which corresponds to the former C = C DB, were found and exhibited good intensities: *m/z* 248.110, 266.121, and 236.127 for position 9 (OA); *m/z* 276.142, 294.152, and 208.115 for position 11 (VA). By the introduction of pyridine rings as ionizable group, the fragments of the omega-end were also detectable in positive mode. Interestingly, for palmitoleic acid (PAL, C16:1n-7, double bond position 9), only one major peak was detected, i.e., the diastereomers coeluted although the carbon chain was only by 2 methylene groups shorter (see Fig. S7). DB position determination was possible by the same carboxy-terminal fragments as for oleic acid: *m/z* 266.120, 248.110 (COOH-end). However, it also shares one fragment with vaccenic acid (*m/z* 208.116, omega-end). This enables the discrimination of PAL from OA and VA on MS1 level and on MS2 level (for MS2 spectra, see Fig. S8).

### Linoleic acid (C18:2n-6,9)

**Fig. 3:**
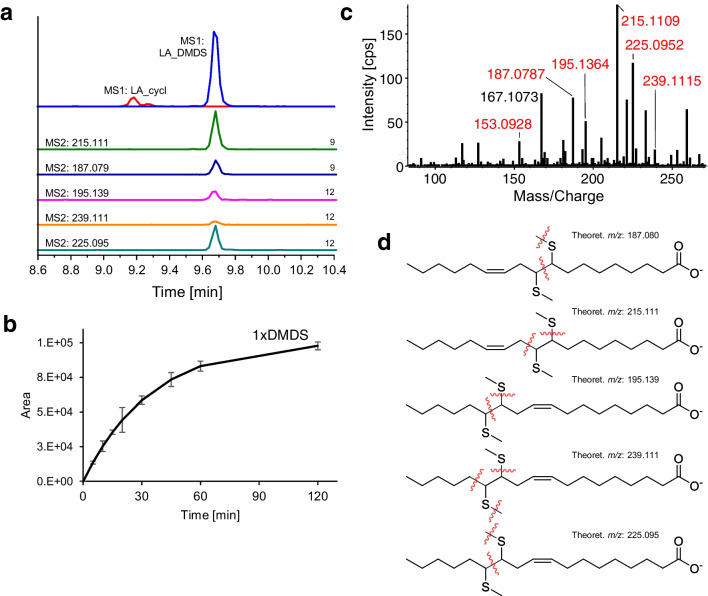
DMDS-derivatized linoleic acid (C18:2n-6,9): **a** MS1-EIC of derivatized linoleic acid (blue; *m/z* 373.224 ± 0.01), cyclic products of linoleic acid (red; *m/z* 405.197 ± 0.01) from TOF–MS scan and MS2-EIC of fragmentation products (40-fold increased for visibility) from PIS (MRM-HR) of mono-derivatized linoleic acid; **b** derivatization kinetics of linoleic acid (fitted, pseudo 1st order, BoxLucas1); **c** MS2 spectrum (CE: 45 V) of derivatized linoleic acid; **d** tentative fragmentation patterns leading to characteristic fragments (position 9: *m/z* 187.080, 215.111; position 12: *m/z* 195.139, 239.111, 225.095)

Due to multiple DBs, the situation gets more complicated for PUFAs. DMDS derivatization of linoleic acid (C18:2n-6,9) leads to mono-derivatized products (Fig. 3a). In spite of isolated DBs, poly-derivatized products were not observed, probably due to the steric hindrance once one DB has been derivatized. This confirms previous observations from GC–MS studies [48, 49]. A small, earlier eluting double peak with the mass of cyclic products was detected. The generation of this unwanted side product is quite low with the used conditions, since short reaction times and low temperature slow down the generation of cyclic products [11]. The determination of DB positions by cyclic products is difficult due to their more complex MS2 spectra [10].

When comparing the derivatization kinetics of oleic acid with linoleic acid, it becomes obvious that linoleic acid needs significantly longer to reach the plateau (Fig. 3b, reaction rate constant *k*: 0.0289 min^−1^, half time *t*_1/2_: 23.97 min).

The MS2 spectrum in Fig. 3c features the fragments for double bond position 9 (*m/z* 187.080, 153.092, and 215.111) and for double bond position 12 (*m/z* 195.139, 239.111, and 225.095). The fragment with *m/z* 187.080 was expected since OA and LA both share a DB at position 9. On the other hand, the fragment with *m/z* 215.111 resulting from α-cleavage on the ω-terminal side is observed with high intensity and must belong to derivatization at position 9 instead of 11 as described for vaccenic acid. Nevertheless, linoleic acid and vaccenic acid can be differentiated by their precursor *m/z* and the presence of fragments with *m/z* 187.080 and 225.095 which are absent in the latter.

DPDS-derivatized linoleic acid shows multiple major peaks with the respective mass (see P1, P2, P3 in Fig. S9), which is due to diastereoisomerism and two derivatization sites (double bond positions 9 and 12). The derivatization kinetics exhibits an exponential increase until 15 min followed by a significant decrease in derivatization product with quite high standard deviations (Fig. S10). The proposed fragmentation patterns are shown in Fig. S9b and c for positions 9 and 12 and MS2 spectra can be found in Figs. S11 and S12. Double bond position 9 can be assigned by fragments with *m/z* 280.136, 266.121, and 262.126, whereas double bond position 12 can be assigned by fragments with *m/z* 288.142, 208.115, and 194.100. This differentiation can be visualized by overlay of MS1-EIC of the precursor with the MS2-EICs of the fragments: Compounds of P1 and P3 are derivatized at double bond position 12, while P2 is derivatized at double bond position 9 (see Fig. S9a). Especially the MS2-EICs allow unequivocal assignments. Nevertheless, the separation of diastereomers and positional isomers of the derivatives leads to increased complexity of the chromatogram and loss of sensitivity. The detection of omega-end fragments allows a differentiation of double bond position 9 in LA from analytes like OA and PAL on MS2 level, as their omega-fragments differ by the additional DB in LA compared to OA.

The further transfer of the DPDS-derivatization to analytes with three DBs was unfortunately not successful due to limited sensitivity and needs further method optimization. The screening of 4,4′-DPDS as reagent was not successful, as it unexpectedly only generated an insignificant amount of fatty acid derivatives.

### Conjugated linoleic acid (C18:2)

Conjugated fatty acids, like conjugated linoleic acids (CLAs), are isomeric with respect to their unconjugated counterparts (in this case linoleic acid). UV detection enables the differentiation of such isomers based on their UV spectra and even enables the identification of double bond configurations [3]. Nevertheless, an unequivocal identification of DB positions without respective standard substances by UV is difficult. However, it is a highly relevant analytical problem since CLAs can be produced during oxidation reactions, e.g., in pharmaceutical formulations [50, 51]. As a proof of principle, 9Z,11Z-CLA (C18:2n-7,9) and 10E,12Z-CLA (C18:2n-6,8), bearing their double bonds in positions 9 and 11 and in positions 10 and 12, respectively, were analyzed.

Interestingly, unlike linoleic acid, both conjugated linoleic acid isomers show two peaks. Since both peaks show the same MS2 spectrum, it can be assumed that diastereomers are separated (Fig. S13). The presence of the DB next to the stereogenic center with the methylthio group seems to favor diastereoselectivity, while the isolation of the two structural elements (DB and methylthio group) by a methylene group in linoleic acid presumably introduces conformational flexibility which prohibits the diastereomer separation. The differentiation of linoleic acid from its conjugated LA isomers is less straightforward since they show similar fragmentation patterns. Nevertheless, 9Z,11Z-CLA can be differentiated from linoleic acid by the absence of the fragment ion with *m/z* 215.111 and the presence of fragment ion with *m/z* 213.095 (Fig. S14a). As expected, the fragments with *m/z* 187.080 and 153.092 are present and allow to pinpoint one double bond in position 9, as discussed for oleic acid and linoleic acid. A possible fragmentation pattern leading to the identification of double bond position 11 is shown in Fig. S14b. It is based on the characteristic product ion with *m/z* 213.095 and additional presence of the less characteristic fragments with *m/z* 225.095 and 239.111.

The DB locations of 10E,12Z-CLA are easier to assign. The double peak elutes slightly earlier (Fig. S13) and shows the fragment ions with *m/z* 227.111 and 239.111 indicating position 12 and the fragment ion with *m/z* 201.095 which is characteristic for position 10 (Fig. S14, c and d). On contrary, the fragments with *m/z* 187.080 and 153.092 are absent. It was reported in the literature for DMDS derivatization of unconjugated C18:2 fatty acids with one cis and one trans configured (isolated) double bond that only the cis double bond will be derivatized, probably due to steric hindrance [48, 49]. Obviously, this is not the case for conjugated fatty acids since 10E,12Z-CLA is trans,cis-configured and DMDS reacted with both DBs.

### α- and γ-Linolenic acid (C18:3)

**Fig. 4:**
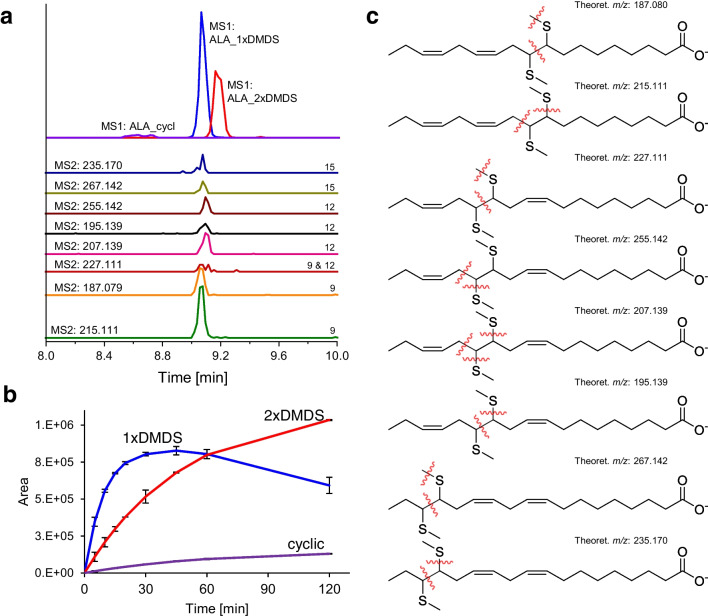
DMDS-derivatized α-linolenic acid (C18:3n-3,6,9): **a** MS1-EIC of mono-derivatized α-linolenic acid (blue; *m/z* 371.208 ± 0.01), bis-derivatized α-linolenic acid (red; *m/z* 465.200 ± 0.01), and cyclic products (purple, *m/z* 403.178 ± 0.01) from TOF–MS scan, MS2-EICs (CE: 45 V) of fragments (300-fold increased: *m/z* 227.111, 235.170, 255.142, 267.142; 100-fold multiplied: *m/z* 195.139, 215.111, 187.080, 207.139) from PIS (MRM-HR) of mono-derivatized α-linolenic acid; **b** derivatization kinetics (fitted, mono-derivatized: ExpGrowDec, di-derivatized: BoxLucas1, cyclic product: BoxLucas1); **c** proposed fragmentation patterns leading to characteristic fragments

α-Linolenic acid (ALA, C18:3n-3,6,9) is one of the most important (essential) ω-3 fatty acids, bearing its DBs on positions 9, 12, and 15. After DMDS derivatization, the mono-derivatized product was used for the assignment of DB positions, but also bis-derivatized and cyclic products were observed (Fig. 4a). The reaction kinetics displays a slow decrease of mono-derivatized products after an initial exponential increase, mainly due to increasing formation of bis-derivatized products (Fig. 4b). DMDS mono-derivatives of all three double bonds can be found. Double bond position 9 can be determined by presence of fragment ions with *m/z* 187.080, 153.092, and 215.111, of which the main fragment ion is *m/z* 215.111 like for linoleic acid. Double bond position 12 is characterized by the fragments with *m/z* 207.139, 195.139, 225.095, and 213.095, while the derivative at the double bond position 15 exhibits fragment ions of *m/z* 267.142 and 235.170. Tentative fragmentation patterns can be seen in Fig. 4c. It must be considered that the fragments for double bond position 15 can also be found in the discussed conjugated linoleic acids and only in low intensities which could be due to steric hindrance during the derivatization reaction. Therefore, for the unequivocal assignment of the fragments to the respective precursor, a chromatographic separation could be helpful which is achieved in the present case, e.g., for α- and γ-linolenic acid and other PUFAs like mentioned CLAs.

Nevertheless, a differentiation between α- and γ-linolenic acid by their MS2 spectra or MS2-EICs of characteristic fragments is possible, since they exhibit completely different fragment ions. γ-Linolenic acid (C18:3n-6,9,12) with double bond position 6, 9, and 12 shows a significantly faster decrease in mono-derivatized product (Fig. S15b) and a double peak in its MS1-EIC, which can be explained with different attachment sites of the DMDS reagent. All 3 DBs can be derivatized to mono-derivatized products. MS2 fragment ions with *m/z* 197.064 and 183.048 can be assigned to double bond position 9, while *m/z* 185.067, 223.079, and 237.095 are presumably derived from derivatives at position 12 (see Fig. S15). For position 6 derivative, some fragments (like *m/z* 142.083) were found to correlate with the first eluting MS1 peak, but a fragmentation pattern could not be suggested. MS2 spectra for derivatized α- and γ-Linolenic acid can be found in Figs. S16, S17, and S18.

### Mixture of polyunsaturated fatty acids

Above experiments were carried out with single standard mixtures. To evaluate applicability to samples in which multiple MUFAs and PUFAs are present simultaneously, it is required that there is adequate specificity of the process, either by LC or MS selectivity. Data were recorded by SWATH acquisition which is a preferential measurement technique in untargeted mass spectrometric analysis for detection of isomers of unknowns in complex samples (as MS2 EICs may be isomer selective) and/or when there is interest in the detection of multiple analytes by targeted MS2 experiments. This is often the case for lipid- or PUFA-containing samples due to their susceptibility to oxidation and further complex degradation reactions [3, 52, 53]. Herein, SWATH-MS was selected to study the reaction products as it allows to enable the extraction of EICs from both precursor and product ions. Retention time overlapping of the EICs of a specific peak group is a good evidence supporting the structural annotations. Overall, the availability of MS2-EIC chromatograms turned out to be helpful for the processing of the data and interpretation of the results.

**Fig. 5:**
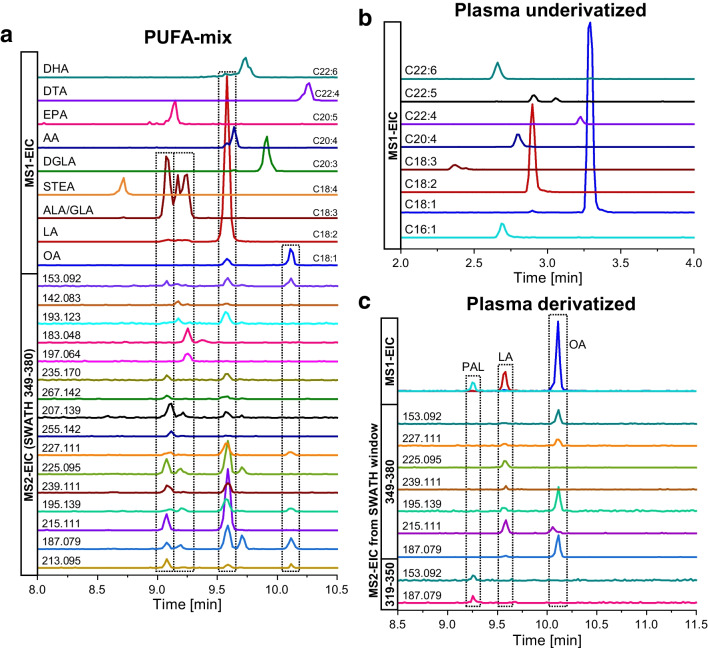
SWATH-MS measurements of DMDS-derivatized PUFA-mix (**a**), underivatized plasma extract (**b**), and DMDS-derivatized plasma extract (**c**). **a** MS1-EIC of mono-derivatized oleic acid (OA, *m/z* 375.239 ± 0.01), linoleic acid (LA, *m/z* 373.224 ± 0.01), α-/γ-linolenic acid (ALA/GLA, *m/z* 371.208 ± 0.01), stearidonic acid (STEA, *m/z* 369.193 ± 0.01), dihomo-γ-linolenic acid (DGLA, *m/z* 399.240 ± 0.01), arachidonic acid (AA, *m/z* 397.224 ± 0.01), eicosapentaenoic acid (EPA, *m/z* 395.208 ± 0.01, multiplied with 10 for better visibility), docosatetraenoic acid (DTA, *m/z* 425.255 ± 0.01, multiplied with 2 for better visibility), and docosahexaenoic acid (DHA, *m/z* 421.224 ± 0.01, multiplied with 2 for better visibility); MS2-EICs (CE: 45 V) of characteristic fragments from SWATH window *m/z* 349–380 (20-fold multiplied: *m/z* 187.080, 215.111, 195.139, 225.095; 50-fold multiplied: *m/z* 239.111, 227.111, 255.142, 207.139, 267.142, 235.170, 197.064, 183.048, 193.123); **b** underivatized plasma extract: MS1-EICs of underivatized C16:1, C18:1, C18:2, C18:3, C20:4, C22:4, C22:5, and C22:6 (assigned with MS-DIAL, but does not allow a statement about double bond positions); **c** derivatized plasma extract: MS1-EIC of derivatized FAs with assigned double bond positions: palmitoleic acid (C16:1), oleic acid (C18:1), and linoleic acid (C18:2) and corresponding MS2-EICs (CE: 45 V) of their characteristic fragments (60-fold multiplied (SWATH window *m/z* 319–350): *m/z* 153.092, 187.080; 40-fold multiplied (SWATH window *m/z* 349–380): *m/z* 227.111, 225.095, 239.111, 195.139, 215.111; tenfold multiplied (SWATH window *m/z* 349–380): *m/z* 187.080). All MS1-EICs are extracted from the TOF–MS scans and MS2-EIC from respective SWATH-MS windows

A commercially available mixture of MUFA and PUFA standards (Polyunsaturated Fatty Acid MaxSpec LC–MS Mixture from Cayman Chemical Company) was derivatized with DMDS and analyzed by the same method (for composition, see Fig. 5a plus docosapentaenoic acid). As discussed above, the different derivatization kinetics suggest varying incubation time optima for different fatty acids; therefore, 30 min was selected as a compromise. The results are depicted in Fig. 5a. The DMDS derivatization of the PUFA-mix showed mono-derivatized products for all contained fatty acids (docosapentaenoic acid (DPA) only shown in Fig. S19 due to very low intensity). It becomes evident from Fig. 5a that the fatty acids with lower number of DBs (LA, ALA/GLA) were observed with higher signal intensities. Although derivatization products of fatty acids with more than three DBs were observed, the unequivocal determination of DB positions was not easily possible due to lack of characteristic fragments and low intensities in MS2 spectra. This might be due to low intensities of mono-derivatized and very high intensities of bis-derivatized products (see Fig. S19), which maybe could be optimized in the future to achieve higher yields of mono-derivatized products.

For C18:1/C18:2/C18:3, the DB positions could be assigned based on MS2-EICs of the characteristic fragments discussed in the chapters above. Figure 5a depicts overlays of MS1 EICs (mono-derivatized FAs) with the MS2-EICs of the respective fragments found in the SWATH window (Q1 precursor isolation window *m/z* 349–380). MS1-EIC of C18:3 showed three peaks, of which the first peak can be assigned to ALA based on aligned MS2 peaks in EICs for the fragments of *m/z* 187.080 and 215.111 for position 9, *m/z* 207.139 and 213.095 for position 12, and *m/z* 235.170 and 267.142 for position 15. The second and third peaks, on contrary, can be assigned to GLA based on retention time of MS2-EICs of fragments with *m/z* 142.083, 183.048, and 197.064. Unfortunately, fragments with *m/z* 223.080 and 237.096 for position 12 are missing which could be, e.g., due to incomplete derivatization or insufficient sensitivity. The presence of linoleic acid (LA) was confirmed by occurrence of fragment ion with *m/z* 187.080 and 215.111 for position 9 and *m/z* 195.139, 239.111, and 225.095 for position 12. The peak of C18:1 was assigned to oleic acid due to the occurrence of the *m/z* 187.080 fragment and absence of any additional fragment peak of *m/z* 215.111 which would correlate to the isomer vaccenic acid.

Dihomo-γ-linolenic acid (C20:3n-6,9,12; double bond positions 8, 11, and 14) similar to ALA and GLA exhibits mono- and bis-derivatized products, whereby the assessment of DB position was made with the mono-derivatized product. Although the intensities were lower when compared to ALA/GLA, a possible assignment of characteristic fragments and MS2 spectra can be found in Figs. S20, S21, and S22.

### Plasma extract

Practical applicability was then tested with plasma. Prior to derivatization with DMDS, the plasma extract was measured underivatized and its free fatty acid profile was determined with MS-DIAL (Fig. 5b). C18:1 and C18:2 fatty acids were observed in high amounts while C16:1, C18:3, C20:4, C22:4, C22:5, and C22:6 fatty acids were only detected at lower levels. After derivatization, mono-derivatized products for C16:1, C18:1, and C18:2 were found (Fig. 5c). C16:1 was assigned to palmitoleic acid due to presence of the fragment with *m/z* 187.079 and 153.092 for position 9 in the SWATH window of *m/z* 319–350. The C18:2 fatty acid was assigned to linoleic acid by occurrence of fragments with *m/z* 215.111 and 187.080 (position 9) and *m/z* 225.095, 195.139, and 239.111 (position 12) in the SWATH window of *m/z* 349–380. C18:1 is mainly assigned to oleic acid due to the major fragment ion of *m/z* 187.080, characteristic for position 9. However, there is a slight fronting which together with the occurrence of fragment ion of *m/z* 215.111 might indicate the presence of vaccenic acid in the plasma extract. This possibility to distinguish isobaric interferences by MS2-EIC in untargeted LC–MS analysis emphasizes the benefits of SWATH acquisition.

## Discussion

Impressive amounts of publications exist which utilize Paternò-Büchi reactions for the determination of DB positions shown in various applications and matrices [13, 15]. Therein, the performance of those methods strongly depends on the selected derivatization reagent and was subsequently optimized, reducing the disadvantages of the initially used reagent (acetone: side reactions, low yield, and *m/z* overlap) [13]. Therefore, we believe that disulfide reagents can be further optimized in a similar manner to allow wider applicability in LC–MS.

The usage of disulfide reagents allows a relatively fast, simple, and cost-effective workflow, due to independence from specific hardware, typically used in some of the other methods for DB position determination (e.g., UVPD, OzID, EAD [17]). Additionally, due to the introduction of sulfur in the derivatized target analytes, characteristic isotope patterns due to ^34^S isotope provide additional information for structural annotation. Further, this method does not need UV light to enable the reaction, which was in the focus for side reactions and potential health risks [17, 54].

DMDS as double bond derivatization agent in LC–MS in combination with SWATH acquisition is a viable strategy to pinpoint DB positions of fatty acids with up to 3 double bonds. However, the assignment of fragment ions and evaluation of MS2 spectra get more difficult with increasing number of DBs. Double bond positions of conjugated fatty acids are less straightforward to distinguish from their unconjugated counterparts. DMDS derivatization of fatty acids is limited to the negative ion mode and provides only characteristic fragment ions from the carboxy-terminal tail. It was shown that DPDS is a viable option to solve this issue, as it allows detection in positive mode and therefore leads to predictable fragmentation which allows straightforward design of two preferred MRM transitions with a characteristic fragment ion from either end of the original DB. The concept can therefore be easily transferred to targeted UHPLC-ESI-QqQ-MS/MS technology with MRM acquisition. The application to fatty acids with more than three double bonds and real samples needs further optimization like the methodology with DPDS as reagent. Further screening of distinct disulfide derivatives like cystine derivatives could be promising as well. Cystine or other functional disulfides allow tuning of the MS properties via amino and/or carboxy group as well as the introduction of specific tags via those reactive groups.

## Conclusions

In this research, we presented the transfer of an established simple and cost-effective method for positional determination of C = C DBs in fatty acids from GC–MS to LC–MS to extend the analyst’s “toolbox” for the comprehensive profiling of fatty acid-containing samples. The derivatization with DMDS in combination with targeted product ion scan (MRM-HR) or untargeted SWATH (MS/MS) detection allowed the straightforward differentiation of DB positions in fatty acids with up to three double bonds. With higher number of double bonds, the differentiation is less straightforward. The applicability of this workflow was also documented for a mixture of PUFAs and for a plasma sample. Derivatization with DPDS introduced pyridine rings into the fatty acids, allowing LC–MS measurements in positive ion mode. It also delivered clearer MS2 spectra, simplifying the evaluation and allowing a better differentiation of LA (double bond position 9) from OA and PAL (double bond position 9) on MS2 level due to detectable fragments of the omega-end. The methodology with DPDS, however, needs further optimization. Derivatization of fatty acids with more than three double bonds was not possible in this preliminary proof of principle evaluation. However, the approach with DPDS indicated the potential for use of tailor-made disulfide-based derivatization reagents to achieve higher derivatization efficiencies, optimized ionization and specific fragmentation, and applicability to fatty acids with more than two double bonds. The applicability of the presented derivatization reagents on larger lipid groups (e.g., glycerolipids, glycerophospholipids) was not tested in this study, but should be possible. Especially DPDS can be advantageous for mono-unsaturated lipids since it is not dependent on the ionization properties of the target molecule. We presented here a proof of principle study on the reactivity of the disulfide agents and their characteristic fragmentations. A follow-up study will address the further optimization in view of real sample application and validation of the methodology.

## Supplementary Information

Below is the link to the electronic supplementary material.

Supplementary file1 (DOCX 1.39 MB)
