# Supplementary material for: Determination of double bond positions in unsaturated fatty acids by pre-column derivatization with dimethyl and dipyridyl disulfide followed by LC-SWATH-MS analysis
Source: Anal Bioanal Chem. 2024 Oct 5;417(13):2753–66. doi: 10.1007/s00216-024-05542-z (PMC12052876; doi:10.1007/s00216-024-05542-z)
Supplement: Supplementary file 1 — Supplementary file1 (DOCX 1.39 MB) [file 216_2024_5542_MOESM1_ESM.docx]

**Supporting Material**

**Determination of double bond positions in unsaturated fatty acids by precolumn derivatization with dimethyl and dipyridyl disulfide followed by LC-SWATH-MS analysis**

Matthias Olfert, Cornelius Knappe, Adrian Sievers-Engler, Benedikt Masberg, Michael Lämmerhofer *

Institute of Pharmaceutical Sciences, Pharmaceutical (Bio-)Analysis, University of Tübingen, Auf der Morgenstelle 8, 72076 Tübingen, Germany

* Author for correspondence:

Prof. Dr. Michael Lämmerhofer

Pharmaceutical (Bio-)Analysis

Institute of Pharmaceutical Sciences

University of Tübingen

Auf der Morgenstelle 8

72076 Tübingen, Germany

T +49 7071 29 78793, F +49 7071 29 4565

E-mail: [michael.laemmerhofer@uni-tuebingen.de](mailto:michael.laemmerhofer@uni-tuebingen.de)

Figure S1: Utilized workflow for DMDS and DPDS sample preparation, followed by LC-MS analysis for double bond position determination. Figure was created with Chemix (<https://chemix.org>).

Table S1: Nomenclature and abbreviations of fatty acids used in this study. Information obtained from LIPID MAPS [1] and „LIPID MAPS Fatty acyl structure level” based on Liebisch et al. [2].

Table S2: Used SWATH-windows in untargeted measurements of MUFA/PUFA-mix and Plasma extract

Figure S2: Mechanism for the derivatization of carbon-carbon double bonds with dimethyldisulfide, proposed by Richter et al. [3].

Table S3: Overview of experimentally observed fragments (*m/z*) and their occurrence for the different DMDS-derivatized fatty acids palmitoleic acid (PAL), oleic acid (OA), cis-vaccenic acid (VA), linoleic acid (LA), α-linolenic acid (ALA), γ-linolenic acid (GLA), 9,11-octadecadienoic acid (9,11-CLA) and 10,12-octadecadienoic acid (10,12-CLA). Here the intensity in the respective sample is visualized in decreasing order: +++ > ++ > + > -. Fields with no entry equal no occurrence of the fragment. Color code: blue: double bond position 6, yellow: double bond position 9, violet: double bond position 10, green: double bond position 11, orange: double bond position 12, red: double bond position 15

Table S4: Suggested transitions (*m/z*) for targeted MRM-Experiments of DMDS-derivatized fatty acids. Color code: blue: double bond position 6, yellow: double bond position 9, violet: double bond position 10, green: double bond position 11, orange: double bond position 12, red: double bond position 15

Table S5: Overview of experimentally observed fragments (*m/z*) and their occurrence for the different DPDS-derivatized fatty acids palmitoleic acid (PAL), oleic acid (OA), cis-vaccenic acid (VA) and linoleic acid (LA). Here the intensity in the respective sample is visualized in decreasing order: +++ > ++ > +. Fields with no entry equal no occurrence of the fragment. Color code: yellow: double bond position 9, orange: double bond position 11, blue: double bond position 12.

Table S6: Suggested transitions (*m/z*) for targeted MRM-Experiments of DPDS-derivatized fatty acids. Color code: yellow: double bond position 9, orange: double bond position 11, blue: double bond position 12.

Figure S3: DMDS-derivatized oleic acid; a: MS1-EIC of mono-derivatized oleic acid (OA) (*m/z* 375.240 ± 0.01 Da) and MS2-EIC of characteristic fragments (for visibility 20-fold increased: *m/z* 187.079, 153.092; 40-fold increased: *m/z* 169.068, 215.111, 213.095); b: proposed fragmentation patterns.

Figure S4: DMDS-derivatized cis-vaccenic acid; a: MS1-EIC of mono-derivatized cis-vaccenic acid (VA) (*m/z* 375.240 ± 0.01 Da) and MS2-EIC of characteristic fragments (for visibility 20-fold increased: *m/z* 215.111; 60-fold increased: *m/z* 243.142, 227.111, 241.127); b: proposed fragmentation patterns.

Figure S5: DMDS-derivatized palmitoleic acid; a: MS1-EIC of mono-derivatized palmitoleic acid (PAL) (*m/z* 347.208 ± 0.01 Da) and MS2-EIC of characteristic fragments (for visibility 100-fold increased: *m/z* 215.111, 213.095, 153.092, 187.068); b: proposed fragmentation patterns.

Figure S6: MS2-spectra of DMDS derivatized palmitoleic acid, a: without zoom, b: with zoom in the relevant section

Figure S7: DPDS-derivatized palmitoleic acid (PAL), detected as [M+H]^+^; a: MS1-EIC of mono-derivatized palmitoleic acid (PAL, *m/z* 475.245 ± 0.01 Da) and MS2-EIC of possible fragmentation products (60-fold increased for visibility); b: Structure of PAL-DPDS derivates and proposed fragmentation patterns.

Figure S8: MS2-spectra of DPDS derivatized palmitoleic acid, a: without zoom, b: with zoom in the relevant section

Figure S9: DPDS-derivatized linoleic acid (C18:2n-6,9), detected as [M+H]^+^; a: MS1-EIC of mono-derivatized linoleic acid (blue; m/z 501.260 ± 0.01 Da) from TOF-MS scan and MS2 EICs (CE: 45 V) of its fragments (50-fold multiplied for visibility) from PIS (MRM-HR) of mono-derivatized linoleic acid; b: tentative fragmentation patterns for double bond position 9; c: tentative fragmentation patterns for double bond position 12

Figure S10: DPDS-derivatized linoleic acid; kinetic study (n=3) displays fluctuations between the replicates and different timepoints.

Figure S11: MS2-spectra of DPDS derivatized linoleic acid (first eluting peak, equals the one of the third eluting peak), a: without zoom, b: with zoom in the relevant section, c: with zoom in the relevant section (smaller mass range).

Figure S12: MS2-spectra of DPDS derivatized linoleic acid (second eluting peak), a: without zoom, b: with zoom in the relevant section, c: with zoom in the relevant section (smaller mass range).

Figure S13: MS1-EIC of DMDS-derivatized 10E,12Z-conjugated linoleic acid (CLA), 9Z,11Z-conjugated linoleic acid (*m/z* 373.224 ± 0.01 Da) and linoleic acid (double bonds position 9 and 12, *m/z* 373.224 ± 0.01 Da).

Figure S14: DMDS-derivatized conjugated linoleic acids (9Z,11Z-CLA; 10E,12Z-CLA); a: MS2 spectrum of derivatized 9Z,11Z-CLA (*m/z* 373.224 ± 0.01 Da); b: tentative fragmentation patterns of 9Z,11Z-CLA leading to characteristic fragments (position 9: *m/z* 187.079, 153.090; position 12: *m/z* 213.095, 239.111, 225.095); c: MS2 spectrum of derivatized 10E,12E-CLA (*m/z* 373.224 ± 0.01 Da); d: tentative fragmentation patterns of 10E,12Z-CLA leading to characteristic fragments (position 10: *m/z* 201.095; position 12: *m/z* 195.139, 227.111, 239.111).

Figure S15: DMDS-derivatized γ-linolenic acid (C18:3n-6,9,12); a: MS1-EIC of mono-derivatized γ-linolenic acid (blue; m/z 371.208 ± 0.01 Da), bis-derivatized γ-linolenic acid (red; m/z 465.200 ± 0.01 Da) and cyclic products (purple, m/z 403.178 ± 0.01 Da) from TOF-MS scan, MS2-EICs (CE: 45 V) of fragments (for visibility 100-fold increased: m/z 197.068, 183.048; 300-fold increased: m/z 142.083, 185.067, 223.079, 237.095) from PIS (MRM-HR) of mono-derivatized γ-linolenic acid; b: derivatization kinetic (fitted, mono-derivatized: ExpGrowDec, bis-derivatized: BoxLucas1, cyclic product: BoxLucas1); c: tentative fragmentation patterns leading to characteristic fragments

Figure S16: MS2-spectra of DMDS derivatized α-linolenic acid, a: without zoom, b: with zoom in the relevant section.

Figure S17: MS2-spectra of DMDS derivatized γ-linolenic acid (first eluting peak), a: without zoom, b: with zoom in the relevant section.

Figure S18: MS2-spectra of DMDS derivatized γ-linolenic acid (second eluting peak), a: without zoom, b: with zoom in the relevant section, c: with zoom in the relevant section (smaller mass range).

Figure S19: EICs of DMDS mono-/bis-derivatized and cyclic products of polyunsaturated fatty acids, from SWATH measurement of PUFA-Mix; a: Stearidonic acid (STEA, mono: *m/z* 369.193, bis: *m/z* 463.184, cyclic: *m/z* 401.163); b: Dihomo-γ-linolenic acid (DGLA, mono: *m/z* 399.240, bis: *m/z* 493.231, cyclic: *m/z* 431.210); c: Arachidonic acid (AA, mono: *m/z* 397.224, bis: *m/z* 491.215, cyclic: *m/z* 429.194); d: Eicosapentaenoic acid (EPA, mono: *m/z* 395.208, bis: *m/z* 489.200, cyclic: *m/z* 427.178); e: Docosatetraenoic acid (DTA, mono: *m/z* 425.255, bis: *m/z* 519.246, cyclic: *m/z* 457.225); f: Docosapentaenoic acid (DPA, mono: *m/z* 423.240, bis: *m/z* 517.231, cyclic: *m/z* 455.210); g: Docosahexaenoic acid (DHA, mono: *m/z* 421.224, bis: *m/z* 515.215, cyclic: *m/z* 453.194); Gaussian smoothed.

Figure S20: DMDS-derivatized dihomo-γ-linolenic acid (DGLA; C20:3n-6,9,12); MS1-EIC of mono-derivatized (*m/z* 399.239 ± 0.01 Da) and di-derivatized DGLA (*m/z* 493.231 ± 0.01 Da) and MS2-EIC of possible characteristic fragments from SWATH-window *m/z* 379-410 (60-fold increased for visibility).

Figure S21: Proposed fragmentation patterns of mono-derivatized dihomo-γ-linolenic acid (DGLA; C20:3n-6,9,12).

Figure S22: MS2-spectra of DMDS derivatized dihomo-γ-linolenic acid (DGLA; C20:3n-6,9,12), a: without zoom, b: with zoom in the relevant section.

Table S7: Equations used in Origin for curve fitting of the derivatization kinetic curves.

| **Analyte and derivatization agent** | **Used equation for curve fitting** | **Variables** |
| --- | --- | --- |
| OA, DMDS derivatized | $f(x)=a*(1-e^{-k*x})$ | *a:* 186065.7  *k:* 0.0723 min^-1^ |
| LA, DMDS derivatized | $f(x)=a*(1-e^{-k*x})$ | *a:* 100918  *k:* 0.0289 min^-1^ |
| ALA, 1xDMDS derivatized | $f\left( x \right)=y_{0}+A_{d}+A_{g}*\left( e^{-\frac{x_{c}}{t_{g}}}-e^{-\frac{x}{t_{g}}} \right);if x\leq x_{c}$  $f\left( x \right)=y_{0}+A_{d}*e^{-\frac{x-x_{c}}{t_{d}}};if x>x_{c}$ | y_0_: 888570  x_c_: 45 min  A_g_:855765  t_g_: 8.85 min  A_d_: -61657.5  t_d_: -47.8 min |
| ALA, 2x DMDS derivatized | $f(x)=a*(1-e^{-k*x})$ | *a:* 1133333  *k:* 0.0203 min^-1^ |
| ALA, cyclic DMDS product | $f(x)=a*(1-e^{-k*x})$ | *a:* 150829  *k:* 0.0164 min^-1^ |
| GLA, 1xDMDS derivatized | $f\left( x \right)=y_{0}+A_{d}+A_{g}*\left( e^{-\frac{x_{c}}{t_{g}}}-e^{-\frac{x}{t_{g}}} \right);if x\leq x_{c}$  $f\left( x \right)=y_{0}+A_{d}*e^{-\frac{x-x_{c}}{t_{d}}};if x>x_{c}$ | y_0_: 397412.9  x_c_: 30 min  A_g_: 348496.6  t_g_: 5.9 min  A_d_: -51081.2  t_d_: -52.7 min |
| GLA, 2x DMDS derivatized | $f(x)=a*(1-e^{-k*x})$ | *a:* 287989.3  *k:* 0.0265 min^-1^ |
| GLA, cyclic DMDS product | $f(x)=a*(1-e^{-k*x})$ | *a:* 40696.9  *k:* 0.0251 min^-1^ |
| OA, DPDS derivatized | $f(x)=a*(1-e^{-k*x})$ | *a:* 177910  *k:* 0.0681 min^-1^ |

**References**

[1] V.B. O’Donnell, E.A. Dennis, M.J.O. Wakelam, S. Subramaniam, LIPID MAPS: Serving the next generation of lipid researchers with tools, resources, data, and training, Science Signaling 12(563) (2019) eaaw2964. <https://doi.org/doi:10.1126/scisignal.aaw2964>.

[2] G. Liebisch, J.A. Vizcaíno, H. Köfeler, M. Trötzmüller, W.J. Griffiths, G. Schmitz, F. Spener, M.J.O. Wakelam, Shorthand notation for lipid structures derived from mass spectrometry, Journal of Lipid Research 54(6) (2013) 1523-1530. <https://doi.org/https://doi.org/10.1194/jlr.M033506>.

[3] N. Richter, J.T. Dillon, D.M. Rott, M.A. Lomazzo, C.T. Seto, Y. Huang, Optimizing the yield of transient mono-dimethyl disulfide adducts for elucidating double bond positions of long chain alkenones, Organic Geochemistry 109 (2017) 58-66. <https://doi.org/10.1016/j.orggeochem.2017.02.003>.
